# Supplementary material for: Neonatal hearing screening among high-risk newborns in Northwestern Nigeria
Source: PeerJ. 2025 Sep 12;13:e20002. doi: 10.7717/peerj.20002 (PMC12435327; doi:10.7717/peerj.20002)
Supplement: Supplemental Information 3 [file peerj-13-20002-s003.docx]

**Questionnaire**

***Questionnaire on Newborn hearing screening in the special care baby unit of Federal Teaching Hospital, Katsina***

Study Number________

**Neonate**

1. Gender
   1. Male
   2. Female
2. Age ……………………days
3. Gestational age at birth ………………….weeks
4. Mode of delivery?
   1. Spontaneous vaginal delivery
   2. Caesarean section
   3. Assisted vaginal delivery
5. Birth weight at delivery……………………….grams
6. Events during SCBU admission
   1. Exposure to ototoxic drugs
   2. Neonatal jaundice requiring exchange blood transfusion
   3. Prolonged mechanical ventilation
   4. Extracorporeal membrane oxygenation
   5. Others (Specify) ………………………
7. Duration of SCBU stay……………………….days
8. Neonatal Sepsis?
   1. Yes
   2. No
9. Family history of hearing loss?
   1. Yes
   2. No
10. Birth Asphyxia (Did the child have difficulties initiating breathing immediately after birth?)
    1. Yes
    2. No
11. Low birth weight
    1. Yes
    2. No
12. Prematurity
    1. Yes
    2. No
13. Head injury
    1. Yes
    2. No

**Mother**

1. Age ………………Years
2. Presence of infections in pregnancy
   1. No
   2. Yes
3. Pregnancy related co-morbidities (Co-existing diseases with pregnancy)
   1. Yes (Specify…………………)
   2. No
4. Ototoxic drug use in pregnancy
   1. Yes
   2. No
5. Consanguinity
   1. Yes
   2. No
6. Mother’s/ Care giver’s concern about hearing, speech, language or developmental delay
   1. Yes
   2. No

**OAE Test**

1. Left ear
   1. Pass
   2. Fail
2. Right ear
   1. Pass
   2. Fail

**AABR Test**

1. Left ear
   1. Pass
   2. Fail
2. Right ear
   1. Pass
   2. Fail

**THANK YOU FOR YOUR PARTICIPATION**
