# Supplementary material for: Neonatal hearing screening among high-risk newborns in Northwestern Nigeria
Source: PeerJ. 2025 Sep 12;13:e20002. doi: 10.7717/peerj.20002 (PMC12435327; doi:10.7717/peerj.20002)
Supplement: Supplemental Information 4 [file peerj-13-20002-s004.docx]

Table S1: Variance Inflation Factors (VIF) for Regression Model

| Predictor Variable | VIF |
| --- | --- |
| Gender | 1.42 |
| Age | 2.25 |
| GA | 9.76 |
| Birth Weight | 15.53 |
| Mothers Age | 1.73 |
| Delivery | 2.4 |
| Neonatal Sepsis | 2.46 |
| Birth Asphyxia | 1.71 |
| LBW | 6.57 |
| Premature | 5.99 |
| Infection during pregnancy | 1.76 |
| Comorbidities | 2.11 |


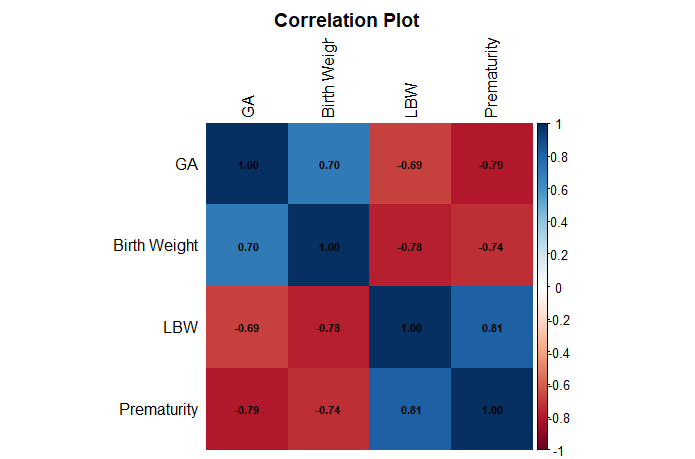


Figure S1. Heatmap of pairwise correlation coefficients between gestational age, birth weight, low birth weight and prematurity.
